# Higher than reported adolescent and young adult clinical trial enrollment during the “Golden Age” of melanoma clinical trials

**DOI:** 10.1002/cam4.1307

**Published:** 2018-02-25

**Authors:** Radhika Sreeraman Kumar, Ram Thapa, Youngchul Kim, Nikhil I. Khushalani, Vernon K. Sondak, Damon R. Reed

**Affiliations:** ^1^ Department of Radiation Oncology Moffitt Cancer Center Tampa Florida 33612; ^2^ Department of Biostatistics and Bioinformatics Moffitt Cancer Center Tampa Florida 33612; ^3^ Department of Cutaneous Oncology Moffitt Cancer Center Tampa Florida 33612; ^4^ Adolescent and Young Adult Program Moffitt Cancer Center Tampa Florida 33612; ^5^ Sarcoma Department Moffitt Cancer Center Tampa, Florida 33612; ^6^ Chemical Biology and Molecular Medicine Program Moffitt Cancer Center Tampa Florida 33612

**Keywords:** AYA, BRAF, clinical trial, immunotherapy, melanoma

## Abstract

Clinical trial enrollments in adolescents and young adults (AYA) with cancer have historically been lower than those in pediatric and older adult populations. We sought to examine therapeutic trial enrollment rates at our cancer center. We performed a retrospective evaluation of AYA patients treated before and after the first checkpoint inhibitor trial opened at our cancer center in 2007. We examined gender, stage at presentation and insurance status in terms of trial enrollment. We compared the trial participation rate of AYA patients with that of older adults. In this adult facility, 12.7% (1,831) of total patients were between age 15 and 39. Overall therapeutic clinical trial rate was 17.6% which increased to 19.8% since 2007. Both nodal disease or metastatic disease at presentation was associated with increasing odds of trial enrollment (OR = 5.36 and *P* < 0.001 for nodal disease and OR = 7.96 and *P* < 0.001 for metastatic disease). There was a nonstatistically significant trend toward improved 3‐year overall survival in the AYA patients with advanced presentation that enrolled on clinical trials compared with those not enrolled on trials since 2007. AYA clinical trial enrollment at a comprehensive care center melanoma program was higher than reported in the literature overall for AYA patients. This 1,831 patient cohort may provide a foundation for more detailed investigation toward quantifying the effects of clinical trial enrollment in terms of age‐specific benefits and toxicities for AYA patients with malignancies that have their peak incidence in older adults.

## Introduction

Adolescent and young adult oncology (AYA) comprises the diagnosis and treatment of 15‐ to 39‐year‐old cancer patients. In comparison to older patients and pediatric patients, AYA patients have had fewer survival improvements than either pediatric or older adult cohorts 1. Among the myriad causes of this disparity are lower clinical trial participation rates than both pediatric and older adult patient cohorts; initially reported to be as low as 1–2% on cooperative group trials 1. The reasons for this being the lowest of any age range are likely multifactorial including suboptimal availability of trials at their location of care, trial awareness among providers and patients, regulatory factors, and a lack of recognition of this unique population of patients when developing therapies and trials 2, 3, 4, 5, 6, 7, 8 When isolated to populations included in pediatric cooperative group trials at National Community Oncology Research Program centers, AYA enrollment on clinical trials is 24%, similar to a single institution study in this population 9, 10. Retrospective data has also demonstrated that adolescent trial accrual is substantially higher, but not as high as the pediatric population, when adolescents are treated at pediatric institutions 11, 12, 13. Clinical trial enrollments in adolescent patients are universally lower than pediatric rates of trial participation and typically in the 10–25% range 11, 12. There is less existing literature focusing on the young adult range of 18–39, with trial rates being typically reported in the 5–10% range (2, 11, 12, 14, 15).

Melanoma is among the most common AYA cancers, constituting 7.9% of all AYA malignancies and 4% of those in adolescent patients 3, 16. The incidence has continued to rise in the adolescent population, and 6.5% of all melanomas are diagnosed before age 34 17, 18, 19. Positive registration clinical trials with checkpoint immunotherapy and MEK/BRAF pathway‐targeted agents in metastatic melanoma have changed a nearly invariably fatal diagnosis to one with significant, even long‐term remissions and improvements in overall survival 20, 21, 22, 23, 24, 25, 26, 27, 28. Young adults, but not adolescents under age 18, had an opportunity to participate in these trials that would have improved their melanoma outcomes 29.

While there have been other studies investigating clinical trial enrollment in the AYA population using both national and regional databases, none have looked at enrollment during this period of successful clinical trials in melanoma. This era is similar to many early acute lymphoblastic leukemia trials, which resulted in survival improvements and defined clinical trials as standards of care in newly diagnosed pediatric cancer patients 30, 31.

H. Lee Moffitt Cancer Center (Moffitt) is a high volume NCI‐designated Comprehensive Cancer Center (NCI‐CCC) with a melanoma center of excellence. The Cutaneous Oncology Program provides care for many pediatric and adolescent referrals along with clinical collaborators, and has a track record of publications in pediatric and AYA melanoma 29, 32, 33. In this paper, we report AYA enrollment into therapeutic melanoma clinical trials at Moffitt from 1986 to 2015 and examine the relevant factors impacting enrollment. As the first checkpoint inhibitor trials began nationwide and at Moffitt in 2007, patients were separated into two cohorts: presentation at Moffitt prior to 2007 and presentation at Moffitt post 2007.

## Methods

Deidentified records of all patients with invasive melanoma treated at Moffitt from 1989 to 2015 were retrospectively reviewed. Demographic information including age at diagnosis, age at presentation, gender, insurance status, stage at diagnosis, stage at presentation to Moffitt, year of diagnosis, vital status, year of death when applicable, and clinical trial enrollment were collected. The AYA cohort encompassed patients who presented to Moffitt between the ages 15–39 with a melanoma diagnosis. Nearly all trials had age 18 as the lower age limit. Patients diagnosed before age 39 who presented to Moffitt after age 40 were classified as older adults. For patients with multiple melanoma diagnoses, the most recent or most advanced stage entries were included. Patients with melanoma who were enrolled on clinical trials for other oncologic diagnoses were identified, coded as not being enrolled on a melanoma clinical trial and excluded from further analysis. Patients were further characterized by type of clinical trial enrollment. Therapeutic trials were defined as involving the use of a systemic therapy while preclinical trials were defined as either banking of tumor tissue or biologic material or registry trials. Trial enrollment was further analyzed by year of presentation.

### Statistical analysis

Rates of clinical trial enrollment were determined by descriptive statistics such as frequency and percentage. Chi‐square test and Fisher's exact test were performed to test for association of each categorical demographic variable with the clinical trial enrollment. Univariate logistic regression analyses were performed to estimate the odds ratio (OR) of the clinical trial enrollment based upon female gender, insured status, local disease and presentation prior to 2007 as reference categories. A multivariable logistic regression analysis was further performed with significant variables from the univariate logistic regression analyses. Kaplan–Meier survival curve estimation and log‐rank test were used to analyze overall survival outcomes. A two‐sided *P* ≤ 0.05 was considered statistically significant.

## Results

From 1986 to 2015, there were 83 open melanoma therapeutic trials: 60 (72.3%) for unresectable metastatic disease, 11 (13.3%) for adjuvant treatment of regional disease, 6 (7.2%) for lymph node evaluation in patients diagnosed with clinically localized disease, and 6 (7.2%) for expanded access to immunotherapeutic drugs for patients with advanced melanoma. Trials were categorized by sponsor, in recognition of the possibility that age limits and other eligibility criteria of relevance to the AYA population might differ between industry sponsored, investigator initiated, and cooperative group sponsored clinical trials. Of the 83 trials, 19 (22.9%) were investigator initiated, an additional 18 (21.7%) were cooperative group/National Cancer Institute sponsored trials, and the remaining 46 (55.4%) were industry sponsored. A total of 14,462 patients with melanoma were included in the analysis. Of these, 1,831 patients (12.7%) were AYA, defined as between the ages of 16 and 39, and presented to Moffitt with either melanoma in situ or invasive melanoma. The characteristics of the 1,740 AYA patients who had at least one invasive melanoma are presented in Table 1. The total number of AYA patients who consented to a clinical trial was 906 (52.6%), of which 610 enrolled in preclinical trials and 306 patients (17.6%) enrolled on therapeutic trials are included in the further analysis (Table 1).

**Table 1:** Characteristics of adolescent and young adult patients enrolled on therapeutic clinical trials

Category	Total cohort with invasive melanoma*N* = 1,740	Patients enrolled in therapeutic trials*N* = 306	Patients not enrolled in therapeutic trials*N* = 1434	*P* a
Gender				
Female	947 (54.4%)	144 (15.2%)	803 (84.8%)	0.005
Male	793 (45.6%)	162 (20.43%)	631 (79.6%)	
Insurance status				
Insured	1099 (63.1%)	203 (18.5%)	896 (81.5%)	<0.001
Medicaid	84 (4.8%)	24 (28.6%)	60 (71.4%)	
Self‐pay	95 (5.5%)	25 (26.3%)	70 (73.7%)	
Uninsured	40 (4.0%)	6 (15%)	34 (85%)	
Unknown	422 (24.2%)	48 (11.4%)	374 (88.6%)	
Stage at Moffitt presentation				
Local	1107 (63.6%)	112 (10.1%)	995 (89.9%)	<0.001
Distant	191 (11.0%)	69 (36.1%)	122 (63.9%)	
Regional 1^b^	22 (1.2%)	4 (18.1%)	18 (81.8%)	
Regional 2^c^	321 (18.4%)	110 (34.3%)	211 (65.7%)	
Unknown	99 (5.6%).	11 (11.1%)	88 (88.9%)	
Presentation at Moffitt				
1986–2006	1028 (59%)	165 (16.1%)	863 (83.9%)	0.051
2007–2015	712 (41%)	141 (19.8%)	571 (80.2%)	

a *P*‐value of chi‐squared test.

b Patients with direct extension into surrounding organs.

c Patients with regional lymph node involvement.

### Gender

Of those on therapeutic trials, 162 (53%) were male and 144 (47%) were female. At Moffitt presentation, 13.6% and 10.3% of male and female patients, respectively, had metastatic disease and 22.0% and 18.1% had regional lymph node involvement. The majority of therapeutic clinical trials included patients with advanced disease, and the number of potential male patients qualifying for therapeutic trials (*n* = 282) was greater than the number of potential female patients (*n* = 269) (OR: 1.43 95% CI: 1.12–1.83, *P* = 0.005).

### Insurance status

Sixty‐six percent of patients on therapeutic clinical trials were insured privately; 7.9% were self‐pay patients; 7.8% were patients on Medicaid; 2% were uninsured; and 16% had an unknown insurance status. Of patients with Medicaid and those paying out‐of‐pocket, 28.6% and 26.3% enrolled in therapeutic trials, compared to 18.5% of their privately insured counterparts (*P *< 0.001).

### Year of presentation

From 1986 to 2006, 16.1% patients enrolled on therapeutic trials. During 2007–2015, 19.8% enrolled on a therapeutic trial. There was both an increase in the number of patients seen per year as well as the number of patients enrolled per year. (Table 1) There were a total of 38 therapeutic trials which opened prior to 2007 and 45 therapeutic trials that opened between 2007 and 2015.

### Stage at Moffitt presentation

Of the 306 patients on therapeutic trials, 36.6% initially presented with localized disease; 22.6% had Stage IV disease on presentation; 35.9% had regional lymphatic involvement; 1.3% had direct extension of their primary tumors; and 3.6% had unknown stage at presentation. Patients with regional nodal disease and distant disease were more likely to enroll in clinical trials, with OR of 4.63 (95% CI: 3.42–6.27) and OR of 5.02 (95% CI: 3.52–7.15), respectively (*P* < 0.001).

### Multivariate analysis

On multivariable logistic regression analysis, AYA patients with regional nodal disease and metastatic disease were more likely to enroll on therapeutic clinical trials than those with localized disease (OR = 5.36 [95% CI: 3.92–7.34] and *P* < 0.001 for nodal disease; OR = 7.96 [95% CI 5.34–11.9] and *P* < 0.001 for metastatic disease). Patients with unknown insurance status were less likely to enroll in clinical trials than patients with insurance (OR = 0.3 [95% CI: 0.2–0.43], *P* < 0.001).

**Figure 1:**
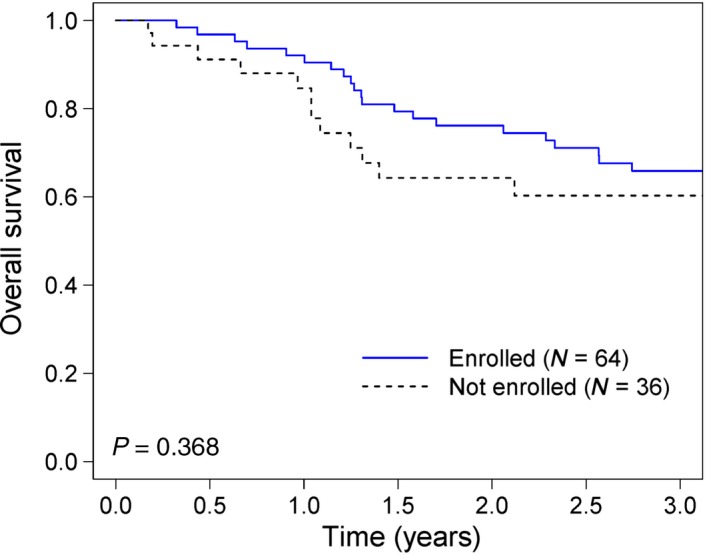
Overall survival of adolescents and young adults with advanced melanoma (nonlocal) enrolled and not enrolled on therapeutic clinical trials from 2007 to 2015.

Among 100 AYA patients with distant metastatic disease, the 3‐year overall survival for the 64 patients who enrolled on clinical trials was better than for those not on trials, but the difference was not statistically significant (log‐rank test *P* = 0.368) (Figure 1).

## Discussion

To our knowledge, our study is the first evaluation of AYA clinical trial enrollment at an adult comprehensive cancer center for a single malignancy. It is different than the body of existing literature, which has focused on the adolescent or young adult malignancies that are more prevalent in pediatric population. We focused on melanoma and the time frames because of the recent improvements in survival on clinical trials leading to FDA approval of eight new agents from 2011 to 2016 34, 35, 36. The overall therapeutic clinical trial enrollment rate found at this Comprehensive Cancer Center of 17.6% is higher than the reported national overall AYA clinical trial enrollment 1, but lower than enrollment at NCORP centers 9, or enrollments in 15‐ to 22‐year‐old patients in centers with an AYA program that has worked to increase trial enrollment 10. Among patients most likely to be eligible for therapeutic trial enrollment, 36% of metastatic patients and 34% of patients with unresectable or regional disease were treated on one of 83 available research protocols, approaching current pediatric oncology participation rates. From 1986 to 2016, there was an increase in the total number of AYA melanoma patients per year as well as an increase in the number of available clinical trials. With 1,740 AYA patients treated for invasive melanoma, our study is also one of the larger cohorts studied in the AYA population. Thirty‐five percent of all AYA patients with melanoma enrolled on a preclinical study as well, which consisted of registry, banking and/or biology studies. This is also an important number as AYA host and disease biology have not been extensively studied and these enrollments may provide a foundation of resources for the research community to improve AYA melanoma care. The rate of AYA patients on clinical trials compares favorably with the 15.7% (1978 enrollments/12,631) therapeutic trial enrollment rate for adults greater than 40 years old with invasive melanoma treated over the same period at our facility.

Our cohort compares favorably with published rates of AYA clinical trial enrollment. However, the population is highly selected. Patients may be referred to this center for the primary purpose of being evaluated for clinical trials. Malignancies with poorer prognoses may lend themselves to increased enrollment due to lack of efficacious standards of care. Melanoma is more likely to affect non‐Hispanic whites, who are more likely than other ethnicities to enroll on clinical trials 37. Thus, our results may not be generalizable to other cancers. The data were also anonymized, so we were not able to analyze the impact of ethnicity or determine the rationale for nonenrollment. Despite the limitations, our results demonstrate that AYA patients did enroll on studies during a period of time when this had a major impact on survival. Noteworthy is that the period of analysis spans the period before the modern AYA movement typically considered around the turn of the century. We entered this investigation with the observation that the Cutaneous Oncology Department was remarkably receptive to AYA program efforts. We hypothesized that this enthusiasm for the care of younger patients would translate to higher than reported clinical trial accruals in this population, and this is indeed what we found. This was an even more appealing question because of the remarkably improved efficacy that these trials afforded patients in the more recent cohort examined. The cancer center's AYA program began in 2011 and so did not contribute to this enrollment formally. While many AYA programs are built from within pediatric oncology, disease‐specific expertise in many AYA cancers resides in medical oncology, and programs built within adult hospitals may be an attractive way of improving AYA participation in clinical trials 38. Our data also highlight the success in AYA enrollment in specimen banking which may inform inherent differences between the biology of a specific malignancy in AYA and adult cohorts.

## Conflict of Interest

None declared.
